# Log In to Experiential Learning Theory: Supporting Web-Based Faculty Development

**DOI:** 10.2196/mededu.7939

**Published:** 2017-09-27

**Authors:** Selma Omer, Sunhea Choi, Sarah Brien, Marcus Parry

**Affiliations:** ^1^ Medical Education Academic Unit Faculty of Medicine University of Southampton Southampton United Kingdom; ^2^ Primary Care and Population Science Academic Unit Faculty of Medicine University of Southampton Southampton United Kingdom

**Keywords:** computer-assisted instruction, models, educational, staff development, education, medical, computer simulation

## Abstract

**Background:**

For an increasingly busy and geographically dispersed faculty, the Faculty of Medicine at the University of Southampton, United Kingdom, developed a range of Web-based faculty development modules, based on Kolb’s experiential learning cycle, to complement the faculty’s face-to-face workshops.

**Objective:**

The objective of this study was to assess users’ views and perceptions of the effectiveness of Web-based faculty development modules based on Kolb’s experiential learning cycle. We explored (1) users’ satisfaction with the modules, (2) whether Kolb’s design framework supported users’ learning, and (3) whether the design principle impacts their work as educators.

**Methods:**

We gathered data from users over a 3-year period using evaluation surveys built into each of the seven modules. Quantitative data were analyzed using descriptive statistics, and responses to open-ended questions were analyzed using content analysis.

**Results:**

Out of the 409 module users, 283 completed the survey (69.1% response rate). Over 80% of the users reported being satisfied or very satisfied with seven individual aspects of the modules. The findings suggest a strong synergy between the design features that users rated most highly and the key stages of Kolb’s learning cycle. The use of simulations and videos to give the users an initial experience as well as the opportunity to “Have a go” and receive feedback in a safe environment were both considered particularly useful. In addition to providing an opportunity for reflection, many participants considered that the modules would enhance their roles as educators through: increasing their knowledge on various education topics and the required standards for medical training, and improving their skills in teaching and assessing students through practice and feedback and ultimately increasing their confidence.

**Conclusions:**

Kolb’s theory-based design principle used for Web-based faculty development can support faculty to improve their skills and has impact on their role as educators. Grounding Web-based training in learning theory offers an effective and flexible approach for faculty development.

## Introduction

### Background

Faculty development is essential for academic staff to develop the pedagogical knowledge and skills that they need to succeed in their teaching roles. Faculty development initiatives can take many forms. Approaches include face-to-face workshops, seminars, short courses, fellowships, and formal qualifications such as postgraduate certificates or master’s degrees [1]. Advances in educational technologies allow learners instantaneous access to resources and tools. Web-based training is rapidly expanding as an approach to faculty development [2]. Options include open-access faculty development resources for clinical teachers in the form of short modules, such as at the London Deanery [3], and online master’s degrees to support physicians to develop skills in education [4]. These demonstrate that Web-based approaches offer several advantages, including convenience and flexibility of learning, access across multiple countries, lower cost, and more time to reflect and learn concepts.

Steinert et al [5] highlighted the need to ground faculty development in theoretical models and principles of teaching and learning to plan, guide, and develop faculty development interventions. Sandars et al [6] also emphasized the importance of grounding work in theoretical models as well as explicitly describing the learning theory when designing technology-supported interactions because this gives an indication of how the technology is intended to facilitate learning and can explain why some e-learning interventions work better than others. Dabbagh [7] wrote about a theory-based design framework to provide a basis for designing e-learning instruction where a pedagogical model (eg, applied learning theory) leads to the specification of instructional strategies (ie, describes techniques that the designer uses to facilitate learning). Technology-enhanced learning or training solutions can then be customized to operationalize the identified instructional strategies.

A limited number of studies described the use of learning theory to guide the design of Web-based resources for professional development. Vrasidas and Zembylas [8] applied a constructivist approach to the development of online resources for teachers’ training. Zhu et al [9] described how a framework can guide the design of augmented reality apps for professional development of general practitioners around the use of antibiotics. These studies showed how learning theory may be used to create the learning environment or to guide learning activities as a substitute for traditional instruction. We did not find a published design framework used for professional development that actually maps the learning activities to a theoretical model or evaluates how the theory-based approach can facilitate meaningful learning and knowledge building.

The University of Southampton’s Faculty of Medicine runs a successful faculty development program designed to meet the needs of the more than 2000 medical teachers who teach basic science and clinical subjects to both undergraduate and graduate entry students. The medical teachers are based in Southampton, across the South of the United Kingdom, the Channel Islands, and more recently in Kassel, central Germany, following the addition of a European bachelor of medicine program. It is therefore difficult for clinicians, especially those based at the more distant hospitals, to attend face-to-face workshops. To improve faculty development opportunities, we developed a blended approach of face-to-face training events and interactive, self-directed, Web-based training, described further in a separate publication [10]. The medical teachers can freely access these modules through a portal called Medical Education Staff Access (MEDUSA). These modules are commonly known as MEDUSA modules.

**Figure 1:**
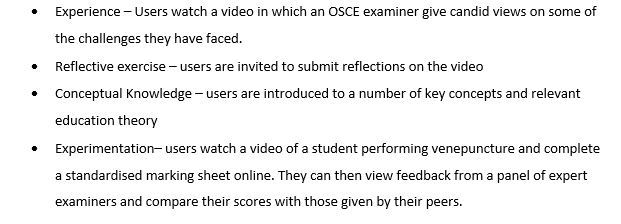
The design of “The role of the OSCE examiner” Medical Education Staff Access (MEDUSA) module based on Kolb’s experiential learning cycle.

**Figure 2:**
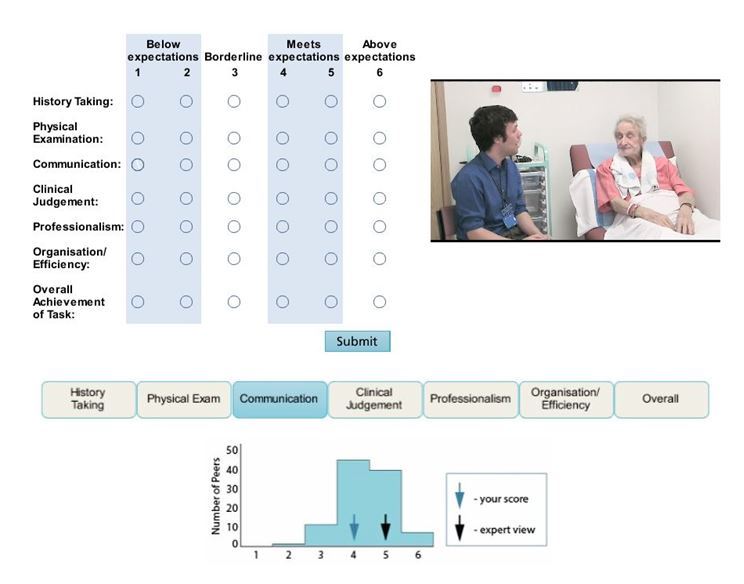
Screenshot from the “Have a go” activity in the module showing a video of a student undergoing an OSCE and the online marking sheet that users complete with the activity feedback that compares their score with expert examiners and peers.

A total of 10 MEDUSA modules have been developed to date, covering a variety of topics ranging from teaching practices (eg, lecturing, giving feedback, supervising student assistantships, and supervising student projects) to assessment (Assessment of Clinical Competence, ACC—formerly the undergraduate mini-CEX—Observed Structured Clinical Exams, OSCEs), raising awareness in issues related to diversity, the student transition from classroom to clinical learning, and the role of the Pastoral Academic Tutor. To ensure maximum flexibility and to enable anytime-anywhere use, the modules were designed to engage learners and to facilitate learning without facilitator inputs. The design of the modules was underpinned by Kolb’s experiential learning cycle [10]. Kolb’s model [11] draws from situated cognition and emphasizes that learning occurs though a four-stage cycle, in which “immediate or concrete experiences” provide a basis for “observations and reflections.” These observations and reflections are distilled into “abstract concepts,” which can be “actively tested,” in turn creating new experiences. The design of “the role of the OSCE examiner” module illustrates this (summarized in Figure 1 and a screen shot from the module in Figure 2).

### Objective

The aim of this paper was to present an educational innovation to emphasize the value of grounding faculty development in theory. We describe a theory-based design approach that we used to guide the design of interactive self-directed e-learning modules for faculty development. On the basis of Kolb’s experiential learning cycle, we created virtual learning environments for feeling, thinking, reflecting, and acting in the modules and evaluated their effectiveness. We studied whether the implemented module design promoted learning through supporting the learning cycle of experience, reflection, conceptualization, and experimentation. Here, we report our findings relating to (1) the users’ satisfaction with the MEDUSA modules and their key design features, (2) how the design features support the users’ ability to learn, and (3) whether the modules affected their work as educators, based on their perceptions after completing the modules. Therefore, the overall aim of this study was to assess users’ views on the effectiveness of designing Web-based faculty development modules based on Kolb’s experiential learning cycle.

## Methods

### Data Collection

Evaluation data were collected from seven MEDUSA modules between March 2010 and July 2013. Ethical approval was waived, as this was an evaluation of an ongoing educational service by a member of staff.

Each module had a built-in evaluation survey that users were invited to complete once they had undertaken the module. The evaluation survey contained a mix of open and closed questions. In the closed questions, users rated each module with regard to its relevance, meeting of learning outcomes, maintenance of interest, amount of interaction, type of interaction, ease of navigation, and overall structure using a 5-point scale (where 1=very dissatisfied and 5=very satisfied). Three open-ended questions asked users to report design features that they found useful in helping them to learn and the features that were not useful and to report how the module had changed their role as educators.

The MEDUSA portal has a learning management function, “My MEDUSA”, which captures the results of each activity to offer users the flexibility of learning at their own pace by allowing them to review previous attempts and to print a summary of their progress and a certificate of completion. Users rated the usefulness of this feature using a 3-point scale (where 1=not useful, 2=useful in parts, and 3=mostly useful).

Users also rated additional design features of fast-track option and discussion forum. Fast track, available in some modules, enables users to move through a briefer learning cycle, with the option to return later for deeper learning by working through the full module. A discussion forum provides opportunities for collaboration with other educators. Users were asked to report whether they used this feature and whether they would use it in other modules.

### Analysis

The responses to closed questions were imported into SPSS, version 22 (IBM Corp, 2013) for analysis by SB. The data were analyzed using descriptive statistics to report the frequency and number of responses.

Responses to open-ended questions were imported into Excel (Windows 2010) for qualitative analysis. Data for each of the three questions were analyzed individually (by SO and SC) using the inductive qualitative approach of content analysis [12]. Responses were analyzed line by line using open coding to systematically develop categories, encapsulating all participants’ views. In content analysis, words or phrases are deemed to reflect important views from participants’ concerns [13]. Illustrative quotations are reported to describe the categories.

## Results

MEDUSA modules were made available to 1365 academic staff and clinicians who teach medical students at the University of Southampton. The modules were promoted through the faculty’s Website, faculty biannual newsletters, and paper leaflets distributed in face-to-face events. Between the period of data collection from March 2010 to July 2013, 284 medical educators (20.8% of total faculty; 50.8% female, 49.2% male) completed 409 modules. Out of the 402 module users with identified roles, 276 (68.6%) were clinical academics, 107 (26.6%) were nonclinical academics, and the remaining 19 users (4.8%) had nonteaching roles, including pastoral and research-only roles. Module evaluation survey was reported on 283 modules of the total 409 completed modules, giving a response rate of 69.1%.

### Satisfaction With MEDUSA Modules

The modules were rated very highly for interactivity, navigation, interest, learning outcomes structure, and relevance with a median score of 4, with over 80% of participants reporting ratings ≥ 4 (satisfied and very satisfied), as shown in Table 1.

The usefulness of the My MEDUSA feature was rated by 255 users (90%). The majority, 161 of 255 users (56.9%), reported it as mostly useful, 84 (32.9%) found it partly useful, and only 10 users (4%) rated it as not useful at all. As the fast-track feature is only available in two modules, only 52 users (18.4%) reported using it, and only 24 users (46.2%) reported that they would recommend using the feature in future. None of the MEDUSA users used the discussion forum.

### Does the Design Framework Support Learning?

Two hundred twenty-five participants made 368 comments about the features that they liked about MEDUSA modules. A total of three themes were identified: the module contents, the delivery modes and technologies, and the structure and presentation of the modules (Table 2).

The module content theme included comments relating to the cases and examples used, key concepts and models introduced, views of students and expert educators, and opportunities for practice and feedback. In the module delivery theme, participants liked the use of multimedia and the design of videos and animations.

We identified a synergy between the design features that the users liked and the four stages of Kolb’s experiential learning cycle used to develop the modules (Table 3). We employed different design features to address the instructional strategies used to operationalize each stage of Kolb’s learning cycle. Participants reflected on features they liked that mapped to the design features at each stage. Examples of some of their quotes are reported in the last column of Table 3.

We used simulations and videos to provide a base for an “experience,” and the participants commented on the usefulness of seeing a video of an OSCE examination and providing them with a real-life example where they can see the interaction between the student and the examiner. We used reflection activities and thought-provoking questions to promote “reflection” in the learning cycle. Although MEDUSA was not designed to give feedback to users on their reflections, My MEDUSA provides a summary of completed activities, enabling users to look back at their reflections after completing the module. Participants generally engaged with reflective activities and commented that the modules provided them with space to think and reflect.

To generate new knowledge through “conceptualization,” we presented theoretical models, video demonstrations for practical tips, and videos and case studies showcasing different perspectives. Participants learned not only from the theoretical models and practical tips presented but also through considering different perspectives from expert educators and students. Participants found it useful to test concepts and experiment through tasks and activities with feedback in each module.

**Figure 3:**
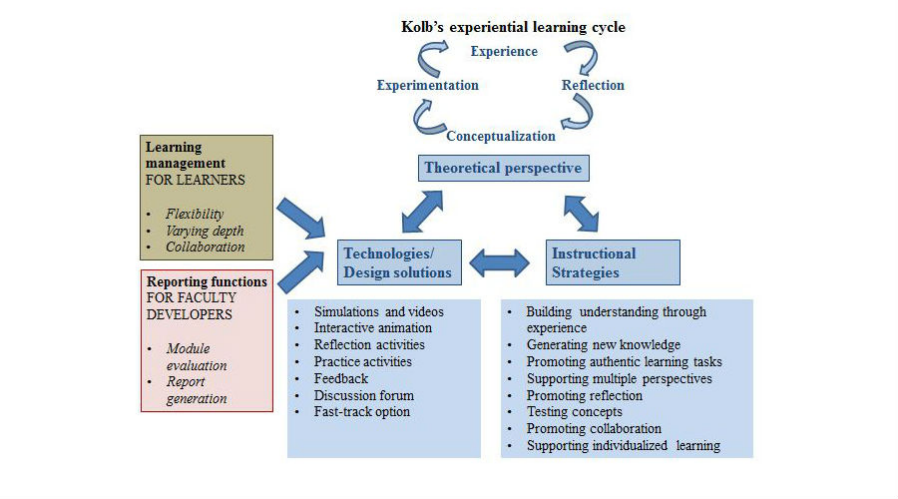
Kolb’s based design framework showing applied in MEDUSA modules showing; instructional strategies and design solutions used (Blue), learning management functions used to meet learner needs (Brown) and reporting functions used to meet the needs of faculty developers/administrators (pink).

Using Kolb’s theory-based design principle for Web-based faculty development enabled us to address our faculty members’ learning needs and meet our organizational needs and constraints (Figure 3).

**Table 1:** Overall satisfaction ratings (evaluation data from seven Medical Education Staff Access (MEDUSA) modules: Assessment of Clinical Competence, Planning and Delivering Lectures, the Role of the OSCE Examiner, Giving Constructive Feedback, the Student Assistantship, Diversity and From Classroom to Clinical Learning. Satisfaction ratings completed by 283 MEDUSA users).

Item	Median rating (1-5 scale)	Rating ≥ 4^a^
Amount of interaction	4	86.7% (241/278)
Ease of navigation	4	82.8% (231/279)
Maintenance of interest	4	81.0% (226/279)
Meeting learning outcomes	4	76.5% (216/282)
Overall structure	4	84.9% (236/278)
Relevance	4	87.8 % (244/278)
Type of interaction	4	83.9% (234/279)

^a^% (n/N), where n reflects the number of rating reporting a score of ≥ 4; and N is the total number of rating (scored between 1-5) reported for that item.

**Table 2:** Themes identified from the Medical Education Staff Access (MEDUSA) features that participants liked (qualitative data from a total of 368 comments reported by 225 participants who completed the evaluation survey).

Themes	Codes
Content	Cases and examples used
	Opportunity to practice
	Feedback on activities
	Practical tips
	Key concepts and models
	Relevant, informative, and realistic
	Relates students and examiner views
	Thought provoking
	Resources and references
Delivery	Animations/video design
	Use of multimedia
	Ease of use, access, and navigate
Presentation	Engaging and interactive
	Appropriate length
	Clear
	Concise
	Simple language
	Organized

**Table 3:** Medical Education Staff Access (MEDUSA) features that were liked by users mapped to Kolb’s experiential learning cycle (qualitative data from a total of 368 comments reported by 225 participants who completed the inbuilt evaluation survey).

Kolb’s experiential learning cycle	Instructional strategies	Technologies/design solutions	Sample quotes
Experience	Building understanding through an experience	Simulations to showcase an experience	“...good to see a video of an examination.”
	Engaging learners in meaningful and relevant tasks so they can apply knowledge in real-world situations	Videos and graphics to bring a case to life or to demonstrate a situation	“...simulated student.”
		Case scenarios describing challenging situations	“...seeing student patient interaction.”
			“...real life example shown.”
Reflection	Promoting reflection	Reflection activities and thought-provoking questions	“...space to consider as well as do.”
			“...it made me think.”
			“...could relate to other examiners description of problems they encountered.”
Conceptualization	Generating new knowledge and concepts	Presenting knowledge and theoretical models though engaging animations	“...animation of Millar’s pyramid.”
	Promoting authentic learning tasks	Interactive case scenarios, video demonstrations for practical tips and guidance	“...examples of constructive feedback.”
	Supporting multiple perspectives	Videos showcasing different perspectives	“...trouble shooting strategies.”
			“...the views of a variety of very experienced lecturers on how to prepare for them and deal with stress involved.”
			“...good to get students views and experiences of feedback.”
			“...video recordings of difficult situations people have encountered.”
Experimentation	Testing concepts through active experimentation	Performing tasks and receiving feedback on task	“...being able to score an actual ACC and compare with peers and experienced examiners.”
	Promoting collaboration and encourage dialogue between teachers and other learners	Discussion forum	“...opportunity to upload my lecture for review.”

**Table 4:** The users reported the various ways in which Medical Education Staff Access (MEDUSA) modules will change their work as educators (Qualitative data from a total of 189 comments reported by 174 participants who completed the inbuilt evaluation survey).

Category	Description (percentage of comments)	Sample quotes	Kolb’s experiential learning cycle
Awareness	Raising awareness, reminding and reinforcing concepts (18%)	“Reinforced some things I knew but do not always focus on and a good opportunity to reflect on own skills and course design.”	Reflection
Knowledge	Gaining knowledge; improved understanding (17%)	“I feel more informed, and have a better idea of standard required.”	Conceptualization
Change	Changing practices—shifting in the focus or method (13%)	“Encouraged me to get students to discuss with each other their feedback after they get it and to offer more opportunity to discuss feedback they get on an assignment.”	Experimentation
Reflection	Making the user reflect on their practice (12%)	“I think it will help me to consider again how I present things to students, to enable as wide an inclusion as possible.”	Reflection
Performance	Building skill, improving performance (11%)	“It will improve how I deliver lectures and help me to keep my audience engaged throughout so that I can maximize how much the students get out of it.”	Experimentation
Confidence	Improving confidence (8%)	“I have more confidence that I’m on the right track!”	Conceptualization
Application	Applying learning into practice (8%)	“I took away some useful ideas to try out with my next student...”	Experimentation

### Do Users Perceive That MEDUSA Will Change Their Work as Educators?

Participants reported the ways in which they anticipated the module would change their work as educators. A total of 174 participants provided 189 open-ended comments. These were categorized thematically into seven categories as shown in Table 4 with illustrative quotes. Participants commented that the modules will enhance their roles as educators by increasing their awareness and knowledge; they also commented that completing the modules helped to improve their confidence and their performance and encourage more reflection in their own practice as well as to consider applying their learning in planning of new teaching practices. Some participants had reported to have even started to implement some changes in their practices.

## Discussion

### Principal Findings

Web-based faculty development was previously shown to be pedagogically promising [2]. Our evaluation shows that the provision of MEDUSA modules in our institution is acceptable to faculty staff with very high rates of satisfaction reported for the modules and with suggestions to improve design features. The findings also provide evidence that the Kolb-based design principle used for Web-based faculty development can support users’ ability to learn and has an impact on their role as educators.

Dabbagh outlined instructional strategies that embody the characteristics of the constructivist views, including activities that promote authentic learning activities, collaboration and social negotiation, promoting articulation and reflection, supporting multiple perspectives, and providing scaffolding [5]. In our theory-based design framework, we embraced these characteristics in the module design and described how to operationalize them through the technologies or design solutions we adopted.

In experiential learning theory, learning is defined as the processing of transformative experiences, which includes concrete experience and abstract conceptualization. The recipients of faculty development are independent autonomous learners who engage in experiences relating to their educational roles. We anticipated that applying Kolb’s learning cycle in the training design would enable them to reflect on these experiences, as well as conceptualize and experiment to motivate behavioral change during the subsequent experiences in the next learning cycle.

Each MEDUSA module was designed based on Kolb’s learning cycle. The module evaluation data suggested that the implemented design features that the users liked in MEDUSA modules directly relate to Kolb’s learning cycle. For example, our users liked design features such as videos that brought cases to life to simulate a relevant *experience* based on Kolb’s model. Similarly, users found the content to be thought provoking, as it stimulated *reflection*. The key concepts and models are related to *conceptualization*. Also among the top ranked were the activities and, in particular, opportunities to practice and get feedback on performance. These promoted active *experimentation* according to Kolb’s model. Our faculty found activities that enabled them to rate student performances and compare their scores with others’ scores particularly helpful. Janick et al used similar Web-based approaches to train faculty to give feedback to students on their performances during small group exercises. They reported that using video clips of student performances and enabling faculty to rate and benchmark their scores increased their ability to assess students and give feedback [14].

Our faculty indicated that completing MEDUSA modules helped them to be better educators through raising their awareness and promoting reflection, increasing their knowledge, and understanding and improving their performance and confidence. Our findings suggest that not only do the stages of Kolb’s learning cycle support learning but different aspects of the learning cycle also become more relevant to individual faculty members for improving their practice and becoming better educators.

We designed our faculty development program to cater to multidisciplinary faculty with diverse backgrounds and with different learning needs. Computer-generated content, such as graphics and videos, can be used to extend and simulate the real-world environment [15]. Therefore, we used simulations in MEDUSA modules to offer those with little or no experience the opportunity to experience authentic tasks, gain knowledge, and practice in a safe environment to prepare them for when they have to do it in real life. Those who have more experience can benefit from improving their performance through reinforcing concepts, gaining different perspectives, and having additional opportunities for practice and feedback. Additionally, we customized some of our modules to acknowledge varying depths of engagement in e-learning through a fast-track option.

With a large portion of our users based at different geographical locations, including overseas, increasing flexibility and access was critical. The learning management function, “My MEDUSA,” enabled flexible learning by enabling users to monitor and review their progress in each module, and the discussion forums provided opportunities for dialogue and collaboration with other educators. Studies have emphasized that one of the benefits of participation in discussion forums is access to an online community of practice [16,17], a network of individuals who share and develop knowledge, values, and experiences and are focused on a common practice and/or mutual goal [18]. Our learners did not take advantage of discussion forums. They accessed modules in their time over 3 years, and they were not likely to return to the module to see whether anyone had commented. Thus, a sense of community did not develop from this feature. Fox et al [19] has suggested that active moderation of discussion groups may be important in increasing communication among participants, but we designed our program to minimize moderation.

On the basis of our experience after implementing Web-based modules for faculty development, we encourage the use of approaches that are grounded in learning theory and that can address their participants’ learning needs and meet their organizational needs and constraints. Future research, using qualitative methodology, could further explore how and why the use of theoretically designed Web-based training enables learning and influences educators in their teaching roles and how to maximize possible features of a learning management system.

### Conclusions

Web-based staff development can provide an effective alternative to traditional face-to-face programs to offer flexibility to geographically dispersed faculty. We have kept educational principles at the core in the development of these e-learning modules for faculty development and based their design on Kolb’s experiential learning cycle. Evaluation of the effectiveness of these modules shows that there is a link between the theoretically informed designed features and what users reported as effective learning. To our knowledge, our approach for mapping learning to different stages of Kolb’s experiential learning cycle is the first of its kind, and our exploratory evaluation supports grounding Web-based faculty development in learning theory.

### Limitations

Further research is required to explore more holistic outcome evaluation of intervention. According to Kirkpatrick’s classification of learning outcomes for educational evaluations [20], our study focused on the level 1 learning outcomes relating to participant satisfaction. Level 2 outcomes relate to testing knowledge, and although participants practiced and applied their learning through activities in each module, we did not test whether the users still retained learning after a certain time had elapsed after the completion of the Web-based training. Future research can address level 3 learning outcomes, that is, whether completing the module can improve teaching.

Although the qualitative findings in this study do provide insight into both what works and what does not, as well as how it can change behavior, there are limitations to open-ended survey questions. A next step could be to conduct an in-depth qualitative study using face-to-face or telephone interviews from a selected sample of participants, which would elicit a more in-depth understanding of how Web-based training can improve educators’ practice.

## Abbreviations

ACC: Assessment of Clinical Competence
MEDUSA: Medical Education Staff Access
OSCE: Observed Structured Clinical Exams
